# DO FAMILY CAREGIVERS MATTER FOR HEALTH CARE UTILIZATION? A STUDY OF MENTAL HEALTH SERVICES USE BY OLDER VETERANS

**DOI:** 10.1093/geroni/igae098.0764

**Published:** 2024-12-31

**Authors:** Mary Wyman, Josephine Jacobs, Lily Stalter, Manasa Ventakesh, Corrine Voils, Ranak Trivedi, Carey Gleason, Byers Amy

**Affiliations:** Veterans Affairs/University of Wisconsin, Madison, Wisconsin, United States; VA Palo Alto Healthcare System, Palo Alto, California, United States; University of Wisconsin Madison, Madison, Wisconsin, United States; University of Wisconsin Madison, Madison, Wisconsin, United States; W.S. Middleton Memorial Veterans Hospital, Madison, Wisconsin, United States; Stanford University/VA Palo Alto Healthcare System, Palo Alto, California, United States; University of Wisconsin Madison, Madison, Wisconsin, United States; VA San Francisco Healthcare System/University of California, San Francisco, California, United States

## Abstract

Mental health (MH) concerns are common in late life. Compared to younger adults, however, older adults are much less likely to use MH services. To guide efforts to increase appropriate utilization, it is important to understand factors impacting MH use. Family caregivers represent a novel, yet understudied, pathway. Using a unique data resource combining 2000-2012 U.S. Health and Retirement Study with Veterans Affairs administrative records, we used mixed effects logistic regression to examine the association between receiving assistance from a caregiver and the likelihood of MH utilization in a sample of community-dwelling, primarily male (96.5%) and non-Hispanic white (77.0%) older veterans. After accounting for demographics, caregiver availability, health factors, and socioeconomic status, caregiving receipt was associated with two-fold odds of MH utilization, compared to receiving no assistance (N=8839 person-observations; OR=2.02; 95% CI 1.54-2.65). The relationship between caregiving receipt and MH use was potentiated by the care recipient both being classified as having dementia and as having less severe MH symptoms. Subsequent logistic regression models examined the impact of multiple caregiver characteristics on utilization among veterans receiving assistance. Primary caregiver female gender was the only predictor which retained an independent, positive association with the outcome (N=764; OR=2.95, CI 1.45-6.00). Findings suggest that caregivers represent an underutilized resource to reduce age-related mental health access disparities, but that certain caregiver subgroups (e.g., male caregivers) may benefit from targeted education or support to help facilitate appropriate MH service use by older adults.

